# Cervical cancer screening and its associated factors among women of reproductive age in Kenya: further analysis of Kenyan demographic and health survey 2022

**DOI:** 10.1186/s12889-024-18148-y

**Published:** 2024-03-08

**Authors:** Zenebe Abebe Gebreegziabher, Birhan Ewunu Semagn, Yitagesu Kifelew, Wondwosen Abey Abebaw, Werkneh Melkie Tilahun

**Affiliations:** 1 Department of Epidemiology and Biostatistics, School of Public Health, Asrat Woldeyes Health Science Campus, Debre Berhan University, Debre Berhan, Ethiopia; 2 Department of Public Health, School of Public Health, Asrat Woldeyes Health Science Campus, Debre Berhan University, Debre Berhan, Ethiopia; 3 Department of Statistics, College of Natural and Computational Science, Oda Bultum University, Chiro, Ethiopia; 4 Department of Epidemiology and Biostatistics, School of Public Health, College of Medicine and Health Sciences, Woldia University, Woldia, Ethiopia; 5 Department of Public Health, College of Medicine and Health Sciences, Debre Markos University, Debre Markos, Ethiopia

**Keywords:** Cervical cancer screening, Associated factors, DHS, Kenya

## Abstract

**Introduction:**

Although cervical cancer screening is one of the most effective strategies to reduce the incidence and mortality of cervical cancer, the percentage of cervical cancer screening in low- and middle-income counties is low. In Kenya, the current nationwide prevalence and associated factors for the detection of cervical cancer is unknown. Therefore, this study aimed to assess the prevalence and associated factors for the detection of cervical cancer screening among women of reproductive age in Kenya using the Kenyan Demographic and Health Survey 2022.

**Methods:**

This study used the most recent Kenyan Demographic and Health Survey data (2022) with a total weighted sample of 16,901 women. A mixed effects logistic regression analysis was performed and in the multivariable analysis, variables with a *p*-value below 0.05 were considered statistically significant. The strength of the association was evaluated using adjusted odds ratios along with their corresponding 95% confidence intervals.

**Results:**

The prevalence of cervical cancer screening in Kenya was 16.81%(95% CI: 16.24, 17.38%). Having a history of abortion (AOR = 1.33, 95% CI: 1.171.50, 1.43), using modern contraceptive methods (AOR = 1.57, 95% CI: 1.25, 1.95), media exposure (AOR = 1.31, 95%CI: 1.03, 1.65), primary education (AOR = 1.56, 95%CI: 1.09, 2.22), secondary education (AOR = 21.99, 95% CI: 1.1.38, 2.87), higher education (AOR = 2..50, 95% CI: 1.71, 3.65), visiting health facility within the past 12 months (AOR = 1.61, 95%CI: 1.46, 1.79), positive HIV status (AOR: 3.50, 95% CI: 2.69, 4.57), being from a community with a higher proportion of educated individuals (AOR = 1.37, 95%CI: 1.13, 1.65) and being from a community with high proportion of poor individuals (AOR = 0.72, 9 5%CI: 0.60–0.87)) were significantly associated with cervical cancer screening.

**Conclusion:**

In Kenya, the prevalence of cervical cancer screening was found to be low. A history of abortion, use of modern contraceptives, exposure to the media, visits to health facilities in the past 12 months, HIV status, level of education, community educational level, and community wealth were identified as significant associated factors for cervical cancer screening. Therefore, it is recommended to implement targeted public health interventions that focus on these identified factors to improve the adoption of cervical cancer screening in Kenya.

**Supplementary Information:**

The online version contains supplementary material available at 10.1186/s12889-024-18148-y.

## Introduction

Cancer is believed to be the second most common cause of death worldwide after cardiovascular disease, with an estimated 9.6 million deaths in 2018 [1]. In 2015, cancer caused 208.3 million disability-adjusted life years (DALYs) globally in both men and women [2]. In women, cervical cancer was predicted to be the fourth most commonly diagnosed cancer and the leading cause of cancer death in 2020, with an estimated 604,000 new cases and 342,000 deaths worldwide [3]. Approximately 85% of these deaths and 90% of cases have occurred in low- and middle-income countries [4]. It affects an estimated 22 million women (over the age of 15) worldwide, accounting for 25.8 to 32% of all female cancers [5]. Cervical cancer is primarily caused by human papillomavirus (HPV). Human papillomavirus encompasses more than 200 distinct strains, with approximately 40 strains commonly infecting the anogenital region. Among these strains, types 16 and 18 are classified as high-risk strains, responsible for almost 70% of all cervical cancer cases [6]. Although cervical cancer is acknowledged as a global issue [7, 8], mounting data show that women in Sub-Saharan Africa (SSA) are more severely affected by the disease [9], and 90% of the 443,000 deaths in general are predicted to occur in SSA in 2030 [10]. The epidemiology and health impact of cervical cancer affect not only women but also their families, communities, and social institutions [11]. The lack of coverage for HPV vaccination could be the potential explanation for the high prevalence of cervical cancer in developing nations [12], and the lower survival rate for cervical cancer in Sub-Saharan Africa (SSA) is primarily attributed to late stage detection and insufficient or delayed access to healthcare facilities [13]. The vast majority of women in SSA decide to visit the hospital after experiencing gynecological symptoms, including abnormal vaginal bleeding, foul smelling vaginal discharge, lower abdominal pain, and hematuria. However, by the time more severe symptoms manifest, it will likely be too late, and the advanced stage of the disease puts the likelihood of survival poor [13, 14].

Although HPV vaccination is recognized as one of the most effective strategies to reduce the incidence of cervical cancer, cervical cancer screening is also a crucial approach to reduce mortality associated with the disease.

Cervical cancer progresses gradually from the precancerous stage to the invasive stage of carcinoma. It may be avoided with screening, which allows for early discovery and the potential for treatment [15]. Cervical cancer screening remains one of the most effective cancer prevention strategies to date. When aberrant changes in cervical epithelial cells are found and treated promptly, the risk of developing cervical cancer will be reduced [16]. It is crucial for early detection of the disease, providing affected women with more treatment options, and increasing the survival rates of cervical cancer survival rates [7]. According to recommendations made by the American Cancer Society (ACS), the World Health Organization (WHO), and the United States Preventive Services Task Force (USPSTF), all eligible women should have a cervical cancer screening at least once every 3 years [17]. There are three main screening approaches for cervical cancer. The first option is HPV testing, which is suggested as the primary screening strategy for women over the age of 30 years. The second procedure is Visual Inspection with Acetic Acid (VIA) or Visual Inspection with Lugol’s (VILI), which is used when HPV testing is not yet available or when there is a danger of loss to follow-up. Finally, the third method is Pap smear, which is recommended in the following situations: a) for women who are not eligible for VIA or VIA/VILI due to the nonvisibility of their squamo-columnar junction (SCJ) and the lack of HPV screening, b) as a primary test for women under 30 years of age, and c) as a co-test with HPV for HIV-positive women [18]. In Kenya, cervical cancer is the second most common malignancy and nine women die from cervical cancer per day, where it is the primary cause of cancer death [19]. Although the government of Kenya has developed a national strategic plan for cervical cancer prevention strategic plan [20], and it is recommended to screen every 5 years for HIV negative individuals and every 2 years for HIV positive individuals [18], the proportion of women screened for cervical cancer was only 19.4% in 2014 [21]. Previous studies reported that lack of knowledge of cervical cancer screening [22], distance to a health facility [21, 23], higher education level [24, 25], media exposure [26, 27], women’s decision autonomy [28], wealth index [21, 25], place of residence [25], number of living children [25], visiting a health facility in the last 12 months [25], age [21, 25], health insurance coverage [25], and region were significantly associated with cervical cancer screening [21].

Few local studies have been conducted in Kenya on cervical cancer screening; however, the current prevalence and associated factors are still unknown throughout the country. Therefore, this study used the Kenyan Demographic and Health Survey (KDHS) 2022 data set to evaluate the current country-wide prevalence of cervical cancer screening and associated variables among Kenyan women of reproductive age.

## Methods and materials

### Study design and sampling procedure

This study was a cross-sectional analysis of secondary data from the Kenyan Demographic and Health Survey carried out in 2022. A nationally representative survey called the DHS gathers information on fundamental health indicators such as mortality, morbidity, use of family planning services, fertility, and maternal and child health. The KDHS chose study participants using a two-stage stratified selection procedure. In the first stage, 1692 clusters were drawn from the Kenya Household Master Sample Frame (K-HMSF) with equal probability and independent selection in each sample stratum. Households were listed in all selected groups, and the resulting list of households served as a sampling framework for the second step of selection, in which 25 households were chosen from each group. The survey includes data sets for men, women, children, births, and households. We used the individual record (IR) file from the women’s data set for this study with a weighted sample of 16, 901 women from 1692 clusters.

## Variables of the study

### Outcome variable

In this study, the outcome variable focused on cervical cancer screening. It was classified into binary format, where a response of “yes” to the question “Have you ever been tested for cervical cancer by health care providers?” was assigned a value of 1, and a response of “no” was assigned a value of 0.

### Independent variables

The study incorporated multiple important independent variables, which have been summarized in S1 Annex.

## Data management and analysis

For valid conclusions, the data were weighted using the sample weight (V005/1000000) before any statistical analysis. Data management and analysis were performed using the STATA version 16 statistical software. Data were extracted, recoded and cleaned and descriptive results were presented as frequencies and percentages. The presence of multicollinearity was assessed after fitting a pseudo-linear regression model with the variance inflation factor (VIF). There were no indicators of multicollinearity because the highest VIF value measured was 3.14, which is within the accepted threshold. The hierarchical nature of DHS data violates the assumptions of independence of observations and equal variance in the traditional logistic regression model. As a result, a mixed effect logistic regression model (fixed and random effect) with a cluster variable (V001) as a random variable was fitted. The presence of a clustering effect was assessed using the intraclass correlation coefficient (ICC), which indicated a significant clustering effect with an ICC value of 15.3%, exceeding the threshold of 10%. The performance of different models: the null model (which does not include any independent variables), the individual variable-only model (which includes individual-level variables such as age and educational status), the community-level-only model (which includes community-level variables like residence and community wealth), and the model with both individual and community-level factors were compared using deviance (− 2 log-likelihood (LL), and the model with the smallest deviance (model with both individual and community-level factors) was chosen as the better model. Chi-square was used to test the presence of a statistically significant association between the outcome variable (cervical cancer screening) and categorical independent variables. The variables that showed a significant association in the chi-square test were selected for inclusion in the multilevel mixed-effect logistic regression model. Additionally, variables with a *p*-value less than 0.2 in the analysis were considered for the multivariable analysis to account for the confounding effects of the variables that passed the analysis. The Wald test was used to obtain *p*-values and variables with p-values below 0.05 were considered statistically significant in the multivariable mixed-effect multilevel logistic regression model. The strength of the association was using the adjusted odds ratio (AOR) along with its corresponding 95% confidence interval (CI).

## Results

### Sociodemographic characteristics of the respondents

This study included a total weighted sample of 16,901 women. The median age at first sexual intercourse was 16 years, with an Interquartile range of ±5. Approximately 36.85% of the respondents identified themselves as Protestant religious followers, while the majority (59.02%) resided in rural areas. Most of the respondents (90.10%) had exposure to at least one form of media. Approximately one fifth of the respondents (23.68%) expressed concern about the distance to health facilities. About a third of the respondents (32.02%) had a positive attitude toward violence with the intimate partner, and 1.99% of the respondents were HIV positive Table 1.

**Table 1:** Sociodemographic characteristics of reproductive age women in Kenya using KDHS 2022

Variables		Weighted frequency (%)
Age	15–24	6256(37.02%)
	25–29	2948(17.44%)
	30–34	2390(14.14%)
	35–39	2314(13.69%)
	40–44	1632(9.66%)
	45–49	1360(8.05%)
Religion	Catholic	3278(19.40%)
	Protestant	6228(36.85%)
	evangelical churches	4064(24.05%)
	African instituted churches	1391(8.23%)
	Islam	1210(7.16%)
	Others	729(4.31%)
Current marital status	Not married	8728(51.64%)
	Married	8173[48.36%]
History of abortion	No	14,731(87.16%)
	Yes	2170(12.84%)
Modern contraceptive use	No	9744(57.66%)
	Yes	7157(42.34%)
Self-reported health status	Bad	431(2.55%)
	Moderate	3281(19.41%)
	Good	13,189(78.03%)
Number of living children	0	4800(28.40%)
	1–2	6024(35.64%)
	3–4	3969(23.49%)
	> = 5	2108(12.47%)
Media exposure	No	1673(9.90%)
	Yes	15,228(90.10%)
Level of education	No education	931(5.51%)
	Primary	6174(36.53%)
	Secondary	6553(38.77%)
	Higher	3243(19.19%)
Distance to the health facility	Not big problem	4002(23.68%)
	Big problem	12,899(76.32%)
Health facility visits within the last 12 months	No	7760(45.92%)
	Yes	9141(54.08%)
Wealth index	Poorest	2628(15.55%)
	Poorer	3007(17.79%)
	Middle	3121(18.46%)
	Rich	3770(22.31%)
	Richest	4376(25.89%)
HIV status	Unknown	2579(15.26)
	Negative	13,986(82.75)
	Positive	336(1.99)
Attitude toward intimate partner violence	Unfavourable	11,490(67.98%)
	Favourable	5411(32.02%)
**Community level variable**		
Proportion of women with media exposure	Low	6831(40.42%)
	High	10,070(59.58%)
Proportion of women with primary and higher education	Low	3605(21.33%)
	High	13,296(78.67%)
Proportion of women with poor wealth index	Low	9339(55.25%)
	High	7562(44.75%)
Proportion of women perceived distance to health facilities as a big problem	Low	9087(53.76%)
	High	7814(46.24%)
Proportion women who had a positive attitude toward intimate partner violence	Low	9492(56.16%)
	High	7409(43.84%)
Residence	Urban	6926(40.98%)
	Rural	9974(59.02%)

### Prevalence of cervical cancer screening among Kenyan women

In this study, the prevalence of cervical cancer screening was 16.81% (95% CI 16.24, 17.38%).

#### Model comparison

Models were compared using deviation, and the model with the lowest deviation was selected. Based on the deviance values, the models showed the following results: the null model had a deviance of 14,620, the individual-only model had a deviance of 12,436, the community-level-only model had a deviance of 14,376, and the model incorporating both individual and community-level variables had a deviance of 12,404. Notably, the model with both individual and community-level variables demonstrated the lowest deviance and was selected as the final model. Therefore, the identification of factors associated with cervical cancer screening was based on the findings of Model III.

### Factors associated with cervical cancer screening among Kenyan reproductive age women using KDHS 2022

In the chi-square test, all categorical independent variables had a statistically significant association with cervical cancer screening at a 5% level of significance and all variables were considered for bivariable analysis. Except for the perceived distance to the health facility, all variables had a *p*-value below 0.2 in the bivariable analysis. In the multivariable mixed effect logistic regression model, the model with both individual- and community-level variables fit the data better than other models; hence, it had the lowest deviance. In the final model, age, religion, history of pregnancy that has ever been terminated, modern contraception use, number of living children, self-reported health status, media exposure, level of education, visit to a health facility within the past 12 months, HIV status, wealth index, educational level of the community, and community wealth index were found to be statistically significant factors associated with cervical cancer screening.

The odds of cervical cancer among women aged 25 to 29 were 1.95 times (AOR = 1.95 95% CI: 1.63, 2.34) higher than those among women aged 15 to 24 years. The odds of cervical cancer screening among women 30–35 years of age were 3.63 times (AOR = 3.63, 95% CI 2.99, 4.41) higher than those among women aged 15 to 24 years. Controlling for other factors, the odds of cervical cancer screening among women 35–39 years were 4.50 times (AOR = 4.50, 95% CI: 3.69, 5.51) higher than those of the age group of 15–24. Holding other factors constant, women 40–44 years had 7.09 times (AOR = 7.09, 95% CI 5.72, 8.80) higher odds of cervical cancer than women aged 15–24. The odds of cervical cancer among women 45–49 years were 6.92 times (AOR = 6.92, 95% CI 5.51, 8.70) higher than those among women aged 15 to 24 years. Regarding religion, respondents with Evangelical church religion had 14% lower odds of cervical cancer screening compared to Catholic religion followers (AOR = 0.86, 95% CI 0.75, 1.00), and Islamic religion followers had 43% lower odds of cervical cancer compared to Catholic religion followers (AOR = 0.57, 95% CI 0.42, 0.78. Having abortion increases the odds of cervical cancer screening by 1.33 (AOR = 1.33, 95% CI: 1.17, 1.50). Using modern contraceptive methods increases the odds of cervical cancer screening (AOR = 1.57, 95% CI 1.25, 1.95). Women who had 1–2 living children had 59% higher odds of cervical cancer screening than women without children (AOR = 1.59, 95% CI 1.32, 1.92). Controlling for other factors, women with 3–4 living children had 1.57 times (AOR = 1.57, 95% CI 1.25, 1.95) higher odds of cervical cancer screening than those without living children. Regarding self-reported health status, women with moderate self-reported health status had 42% lower odds of cervical cancer screening compared to women with poor self-reported health status (AOR = 0.58, 95% CI 0.44, 0.76), and women with good self-reported health status had 48% lower odds of cervical cancer screening compared to women with poor self-reported health status (AOR = 0.52, 95% CI 0.39, 0.67). Media exposure increased the odds of cervical cancer screening by 31%(AOR = 1.31, 95% CI: 1.03, 1.65). Regarding educational status, women with primary education had 56% higher odds of cervical cancer screening (AOR = 1.56, 95% CI: 1.09, 2.22), women with secondary education had 1.99 times (AOR = 1.99, 95% CI: 1.38, 2.87) higher odds of cervical cancer, and women with higher education had 2.50 times (AOR = 2.50, 95% CI: 1.71, 3.65) higher odds of cervical cancer screening than women with no education. Visiting a health facility within the last 12 months increased odds of cervical cancer screening by 61% (AOR = 1.61, 95% CI: 1.46, 1.79). Wealth index has a direct association with cervical cancer screening: Women with a poorer wealth index had 27% higher odds of cervical cancer screening (AOR = 1.27, 1.03, 1.57), women with richer wealth had 30% higher odds of cervical cancer screening (AOR = 1.30, 95% CI: 1.02, 1.66), and women with the richest wealth had 1.57 times (AOR = 1.57, 95% CI: 1.19, 2.06) higher odds of cervical cancer screening than women with the poorest wealth. HIV positive individuals had 3.5 times (AOR = 3.5, 95%CI: 2.69, 4.57) higher odds of cervical cancer screening than HIV negative individuals. Regarding community-level factors, women in a community with a high proportion of poor individuals had 28% (AOR = 0.72, 95% CI 0.60, 0.87) lower odds of cervical cancer screening than women in a community with a low proportion of poor individuals, and women in a community with a high proportion of educated individuals had 37% higher odds of cervical cancer screening than women in a community with a low proportion of educated individuals (AOR = 1.37, 95% CI: 1.13, 1.65) (Table 2).

**Table 2:** Bivariable and multivariable multilevel logistic regression analysis for cervical cancer screening and its associated factors among reproductive age women in Kenya using KDHS 2022: In the multivariable analysis, adjustments were made for variables that demonstrated a significant association in the bivariable analysis

Variables		Null model	Model I		Model II	Model III		
		AOR(95%CI)	AOR (95% CI)		AOR (95% CI)	COR (95% CI)	AOR (95% CI)	*P*-value
Age	15-24		1			1	1	
	25-29		1.96(1.63, 2.35)			3.40(2.89, 3.99)	1.95( 1.63 - 2.34)	0.001*
	30-34		3.68(3.03, 4.46)			6.02(5.13, 7.07)	3.63(2.99 - 4.41)	0.001*
	35-39		4.57(3.74, 5.59)			7.28(6.21, 8.53)	4.50(3.69 - 5.51)	0.001*
	40-44		7.24(5.84, 8.98)			10.80(9.14, 12.77)	7.09(5.72 - 8.80)	0.001*
	45-49		7.09(5.65, 8.90)			10.05(8.41, 12.00)	6.92( 5.51 - 8.70)	0.001*
Religion	Catholic		1			1	1	
	Protestant		0.90(0.79, 1.02)			0.96(0.85, 1.09)	0.90 (0.79 - 1.02)	0.105
	Evangelical churches		0.86(0.75, 0.99)			0.89(0.78, 1.02)	0.86 (0.75 - 1.00)	0.048*
	African instituted churches		1.13(0.93,1.38)			1.11(0.93, 1.32)	1.13( 0.93 - 1.37)	0.211
	Islam		0.54(0.40, 0.73)			0.32(0.24, 0.42)	0.57 (0.42 - 0.78)	0.000*
	Others		1.07(0.82, 1.39)			0.90(0.71, 1.15)	1.09 (0.84 - 1.42)	0.517
Marital status	Not married		1			1	1	
	Married		0.88(0.7, 0.98)			1.89(1.73, 2.08)	0.90(0.81 - 1.00)	0.056
History of abortion	No		1			1	1	
	Yes		1.32(1.17, 1.50)			2.23(1.99, 2.51)	1.59(1.32 - 1.92)	0.001*
Modern contraceptive use	No		1			1	1	
	Yes		1.30(1.18, 1.92)			1.89(1.73, 2.06)	1.57(1.25 - 1.95)	0.001*
Self-reported health status	Bad		1			1	1	
	Moderate		0.58(0.44, 0.77)			0.71(0.54, 0.93)	0.58(0.44 - 0.76)	0.001*
	Good		0.51(0.39, 0.67)			0.42(0.32, 0.54)	0.52(0.39 - 0.67)	0.001*
Number of living children	0		1			1	1	
	1-2		1.59(1.32, 1.92)			4.90(4.22, 5.70)	1.59(1.32 - 1.92)	0.001*
	3-4		1.55(1.24, 1.94)			6.99(5.98, 8.17)	1.57(1.25 - 1.950	0.001*
	>=5		1.22(0.94, 1.58)			5.49(4.56, 6.60)	1.26(0.97 - 1.64)	0.081
Media exposure	No		1			1	1	
	Yes		1.35(1.07, 1.69)			2.29(1.87, 2.80)	1.31(1.03 - 1.65)	0.024*
Level of education	No education		1			1	1	
	Primary		1.89(1.45, 2.64)			3.04(2.24, 4.13)	1.56(1.09 - 2.22)	0.014*
	Secondary		2.40(1.70, 3.41)			2.43(1.78, 3.30)	1.99(1.38 - 2.87)	0.001*
	Higher		2.98(2.07, 4.28)			4.50(3.29, 6.16)	2.50(1.71 - 3.65)	0.001*
Age at first sex	Median 16±5(IQR)					1.10(1.09, 1.11)	1.01(1.00 - 1.03)	0.042*
Health facility visits within the last 12 months	No	1			1		1	
	Yes	1.61(1.46, 1.65)			1.95(1.77, 2.15)		1.61(1.46 - 1.79)	0.001*
Wealth index	Poorest	1			1		1	
	Poorer	1.33(1.09, 1.65)			1.72(1.42, 2.10)		1.27(1.03 - 1.57)	0.026*
	Middle	1.40(1.14, 1.73)			2.06(1.71, 2.49)		1.22(0.98 - 1.52)	0.073
	Richer	1.62(1.30, 2.01)			2.55(2.12, 3.07)		1.30(1.02 - 1.66)	0.034*
	Richest	2.04(1.63, 2.57)			3.50(2.90, 4.23)		1.57(1.19 - 2.06)	0.001*
Attitude toward intimate partner violence	Unfavourable	1			1		1	
	Favourable	0.91(0.81, 1.01)			0.76(0.69, 0.84)		0.91(0.81 - 1.03)	0.125
HIV status	Negative	1			1		1	
	Unknown	2.10(1.62, 2.72)			0.15(0.12, 0.18)		0.48(0.37, 0.63)	0.00*
	positive	7.48(5.20, 10.75)			5.17 (4.03, 6.63)		3.50(2.69, 4.57)	0.00*
Proportion of women with media exposure		Low	1	1	1		1	
		High	1.35(1.18, 1.55)		1.93(1.70, 2.19)		1.11(0.96 - 1.29)	0.164
Proportion of women with primary and higher education		Low	1	1	1		1	
		High	1.95(1.65, 2.30)		2.42(2.05, 2.84)		1.37(1.13 - 1.65)	0.001*
Proportion of women with poor wealth index		Low	1	1	1		1	
		High	0.57(0.48, 0.67)		0.48(0.43, 0.55)		0.72(0.60 - 0.87)	0.001*
Proportion of women perceived distance to health facilities as a big problem		Low	1	1	1		1	0.621
		High	1.04(0.92, 1.19)		0.75(0.66, 0.85)		1.04(0.90 - 1.19)	0.683
Proportion of women who had a positive attitude toward intimate partner violence		Low	1	1	1		1	
		High	0.94(0.82, 1.06)		0.71(0.62, 0.80)		1.01(0.88 - 1.17)	0.839
Residence		Rural		1	1		1	
		Urban	1.06(0.91, 1.24)		0.68(0.60, 0.77)		1.03(0.86 - 1.23)	0.76

* Indicates statistically significant at a 5% level of significance based on the Wald test results

## Discussion

In this study, the prevalence and associated factors of cervical cancer screening among reproductive-age women in Kenya were assessed. The prevalence of cervical cancer screening in Kenya was 16.81%(95% CI: 16.24, 17.38%). Age, religion, history of abortion, modern contraception use, number of living children, self-reported health status, media exposure, level of education, visits to health facilities within the past 12 months, wealth index, level of education in the community educational level, community media exposure and community wealth index were significantly associated with cervical cancer screening.

In the current analysis, the prevalence of cervical cancer screening among reproductive age women in Kenya was 16.81% (95% CI 16.24, 17.38%). This prevalence is higher than that of a study conducted in Ethiopia (14.79%) [29], and a study conducted in Oman (15.7%) [30], but it is lower than the prevalence of a previous Kenyan DHS 2014 study (19.4%) [21], Malaysia (48.9%) [31], Cameron (55.7%) [32], Kathmandu (31.6%), Rwanda (28.3%) [33] and it is also lower than the average coverage of cervical cancer screening coverage in developed countries (63%) [34]. This might be due to the difference in wealth status, educational status, and exposure to the media. Cultural variations might also contribute to the observed discrepancy, as certain cultural norms impose restrictions on women’s access to health facilities based on cultural standards. These norms may cause women to perceive cervical cancer screening as a taboo topic, which may reduce the use of screening programs [35]. Additionally, financial barriers could be a possible explanation for the lower prevalence of cervical cancer screening in Kenya compared to developed countries. Despite the availability of free cervical screening services in many sub-Saharan African countries, the utilization of these services may be impeded by out-of-pocket payments and concerns about hidden charges.

The odds of cervical cancer screening among older women were higher than among younger women. This is consistent with a study conducted in South Africa’s general population [36, 37]. One possible explanation is that older women may have more frequent visits to healthcare facilities for purposes such as antenatal care and postnatal care. This increased contact with healthcare providers offers opportunities for them to receive counseling and information about cervical cancer screening services. In addition, older women may perceive themselves to be at greater risk of developing cervical cancer, which can increase their motivation to undergo screening.

Regarding religion, respondents with followers of the evangelical church religion and the Islamic religion had lower odds of cervical cancer screening than those of the catholic religion. Cultural norms and modesty concerns can discourage women from accessing preventive healthcare services involving intimate inspections, influencing their desire to carry out cervical cancer screening.

Women with a history of abortion had higher odds of cervical cancer screening than those without it. One possible explanation is that women who have had abortions may have had previous contact with healthcare professionals, which may have increased their awareness of the importance of frequent health checks, including cervical cancer screening. Furthermore, the experience of having an abortion may have improved women’s knowledge about reproductive health issues and the need for preventative measures, pushing them to seek cervical cancer screening proactively.

Using modern contraceptive methods increases the odds of cervical cancer screening. This is in line with the study conducted in five Sub-Saharan African countries [25] and a study conducted in Burkina Faso [38]. One potential reason could be that individuals who utilized modern contraception had a higher probability of meeting healthcare professionals on a regular basis. During these interactions, healthcare professionals might have taken the opportunity to provide information about screening and treatment services to their clients.

Women who have a larger number of children are more likely to undergo cervical cancer screening. This may be attributed to the fact that women with more children tend to have more regular interactions with healthcare professionals during important stages such as the preconception period, antenatal care visits, childbirth, and postpartum period. These frequent interactions with healthcare professionals provide them with opportunities to receive information about cervical cancer screening, and they are more likely to use screening services during their preconception and postnatal periods.

Women with moderate and good self-reported health status had lower odds of cervical cancer screening than women with poor self-reported health status. One possible explanation is that women who report a good self-perceived health status may perceive themselves to be at a lower or no risk of developing cervical cancer. As a result, they may be less inclined to prioritize or seek cervical cancer screening. On the other hand, women who report a poor self-perceived health status may suspect cervical cancer as a potential cause of their health problems. This awareness or suspicion could motivate them to screen for cervical cancer and seek appropriate health services.

Exposure to the media increases the odds of cervical cancer screening. This is consistent with a study conducted in Namibia [27]. One potential reason is that women who are exposed to the media are more likely to receive information about the health and financial advantages of cervical cancer screening. Furthermore, media exposure can contribute to greater awareness and understanding of global trends and perspectives, enabling women to distinguish between cultural taboos and factual information. This increased knowledge and discernment can foster a positive attitude toward the use of healthcare services, including the detection of cervical cancer.

Women with primary, secondary and higher education had higher odds of cervical cancer screening than those of uneducated women. This finding is supported by a study conducted in Latin America among women with low and middle income [39], a study conducted in China [40], and a meta-analysis conducted in developed countries [41]. One possible explanation is that women with higher levels of education are more likely to engage with maternal health services and undergo gynaecologic examinations. This increased engagement provides them with regular opportunities to access obstetric and gynecologic care in healthcare institutions. Additionally, educated women have the ability to read and comprehend information about cervical cancer screening from healthcare institutions and the media, which can influence their attitudes towards screening. Additionally, educated women are often less influenced by cultural taboos and possess greater autonomy compared to individuals with lower levels of education. This increased autonomy enables them to make informed decisions about their healthcare, including the choice to undergo a cervical cancer screening.

Visiting a health facility within the past 12 months increases the odds of cervical cancer screening. This is consistent with a study conducted in Latin America [42]. One possible explanation is that healthcare providers may distribute knowledge about cervical cancer preventive measures and encourage screening [43]. Furthermore, screening may overlap with pre- or post-treatment of other health conditions. Furthermore, attending a health facility can increase the chances of a woman to obtain health insurance, and cervical cancer screening can be one of the services covered by health insurance [43, 44].

Women with the highest wealth had higher odds of cervical cancer screening. This finding is supported by a study conducted in Latin America [39, 42]. One potential reason is that women with higher financial resources encounter fewer obstacles to accessing cervical cancer screening. Factors such as household responsibilities and transportation limitations may pose fewer challenges for women with more financial means. They have the financial freedom to seek screening services in private or public health facilities, as necessary. Additionally, the economic freedom enjoyed by women with a high wealth index may contribute to their trust in cervical cancer therapy, even if positive outcomes are achieved. Their financial stability may provide them with a sense of security and confidence in the effectiveness of treatment options, leading to a more favourable attitude towards screening and therapy utilization [42].

Women in a community with a high proportion of educated individuals had higher odds of cervical cancer screening than women in a community with a low proportion of educated individuals. In communities where a significant proportion of individuals are educated, cervical cancer screening may be viewed as a routine and essential practice rather than a taboo. Additionally, women from such communities may experience less fear regarding screening results, as they believe their community will provide understanding and support, even in the case of a positive diagnosis for cervical cancer.

## Strengths and limitations of the study

Regarding strengths, the study was based on a large, weighted and nationally representative dataset that might have adequate statistical power to detect true associations. Furthermore, to obtain a reliable standard error and estimate taking into account the clustering effect, an advanced model (multilevel mixed effect) was used. However, the study is not without limitations. First, the study relies on self-reported screening history without specific date, potentially leading to overestimation of effect size. Second, the questionnaire only captures lifetime experiences without specifying screening methods, which could impact effect size due to variations in sensitivity and specificity. Third, due to the cross-sectional nature of the data, it is not possible to determine temporal relationships. Fourth, the unavailability of human papillomavirus (HPV) vaccination status prevented us from determining whether participants were protected solely by vaccination or if they were protected by both vaccination and other preventive measures. Fifth, the survey focused on the screening history of the respondents, but did not inquire about the specific screening methodologies used, which can vary in terms of sensitivity and specificity. Additionally, the survey did not specify the year of screening, meaning individuals who were screened 10 years ago could be categorized as screened without knowing their current screening status. Finally, there is a potential for social desirability bias as the data were collected through face-to-face interviews.

## Conclusion

The prevalence of cervical cancer screening in Kenya was low. Age, history of abortion, modern contraceptive use, number of living children, exposure to the media, visits to health facilities in the past 12 months, wealth index, community educational level, and exposure to the community media had positive associations with cervical cancer screening. However, being married, self-reported good and moderate self reported health status, and community wealth were negatively associated with cervical cancer screening. Therefore, to increase cervical cancer screening uptake in Kenya, public health interventions targeting uneducated women, those in the poorest wealth, those who did not use modern contraceptive methods, those who did not receive media exposure and those who did not visit a health facility in the previous 12 months. To achieve this, targeted awareness campaigns can be initiated in low-income areas, emphasizing the importance of cervical cancer screening. Collaborations with local clinics, community health workers, and organizations can be established to provide information and reminders regarding screening. Additionally, organizing “screening open days,” mobile clinics, and outreach programs, along with HPV vaccination campaigns, is also recommended.

### Supplementary Information

**Additional file 1: Table S1.** Independent variable, and their categorization for cervical cancer among reproductive age women in Kenya, 2022.

## Data Availability

The datasets used and / or analyzed during the current study are available online on the DHS measure website http://www.dhsprogram.com.

## Abbreviations

AIC: Acacia information criteria
AOR: Adjusted odds ratio
CI: Confidence interval
ICC: Intraclass correlation coefficient
IPV: Intimate partner violence
KDHS: Kenyan demographic and health survey
SSA: Sub-Saharan Africa, (ICC)
