# Supplementary material for: Cervical cancer screening and its associated factors among women of reproductive age in Kenya: further analysis of Kenyan demographic and health survey 2022
Source: BMC Public Health. 2024 Mar 8;24:741. doi: 10.1186/s12889-024-18148-y (PMC10921781; doi:10.1186/s12889-024-18148-y)
Supplement: Supplementary file 1 — Additional file 1: Table S1. Independent variable, and their categorization for cervical cancer among reproductive age women in Kenya, 2022. [file 12889_2024_18148_MOESM1_ESM.docx]

Table S1: Independent variable, and their categorization for cervical cancer among reproductive age women in Kenya, 2022

| Variable | Categorization | |
| --- | --- | --- |
| Age | “15-24=1, “25-29”=2,”30-34”=3, “35-39”=4, “40-44”=5, “45-49”=6) | |
| Religion | "Catholic"=1, "protestant"=2, "Evangelical churches"=3, "African instituted churches"=4, "Islam"=5, and the remaining was coded as 6 "others") | |
| Residence | “Urban”=1, “rural”=2 | |
| level of education | “No education”=0,“primary”=1, “secondary”=2,& “higher”=3 | |
| Number of living children | In this study, it was categorized into four categories ("0"=0, "1-2"=1, "3-4"=2, "5/maximum"= 3) | |
| Media exposure | Individuals who had encountered at least one form of media exposure (such as radio, television, newspaper, or internet) were classified as "exposed" with a value of 1. On the other hand, individuals who had no exposure to any form of media were categorized as "non-exposed" with a value of 0. | |
| Distance to the health facility | Based on the information provided in the DHS dataset, individuals who consider distance to a health facility as a significant problem are categorized as "1" while those who do not perceive distance to a health facility as a problem are categorized as "0." The categorization is not determined by the actual physical distance, but rather by individuals' subjective perception of the distance. | |
| Health facility visits within the past 12 months | Individuals who underwent screening or treatment for another disease at a healthcare facility within the last 12 months were assigned a categorical value of "1" (yes), while those who reacted negatively were assigned a value of "0" (no). | |
| Wealth index | It is a composite variable that is measured by different variables like having TV and it was categorized into five (1“poorest”, 2 “poorer”, 3 “medium”, 4 “rich”, and 5 “richest)” | |
| History of abortion | It is used exactly as it is shown in the dataset (v228). Have you ever terminated a pregnancy? (0 “no”, 1 “yes”). | |
| Modern contraceptive use | In the original dataset, it was presented in four categories: 0 "no method" 1 "folkloric method "2 "traditional method" and 3 "modern method". In this study, it was re-categorized into two 1 " modern contraception users, and the rest were coded as 0 "not modern contraception users" | |
| Self-reported health status | In the DHS dataset it was presented from 1 "very good" to 5 "very poor" For this study it was categorized into 3 by recoding very bad and bad into 0 "bad", 1 "moderate", very good, and good 2 "good". The evaluation of health status is reliant on individuals' subjective perceptions rather than objective measures of their actual health status. | |
| Residence | 0 “urban”, 1 “rural” | |
| **Community-level variables that were aggregated from individual-level variable** | | |
| IPV | | \| The study examined women's justification of intimate partner violence (IPV) towards themselves. This was assessed by asking women whether they believed a man had the right to physically harm his wife in five hypothetical scenarios: (1) if she leaves without informing him; (2) if she neglects the children; (3) if she argues with him; (4) if she refuses to have sexual relations with him; and (5) if she does not properly prepare meals. Based on individual responses, women were categorized as having a favorable attitude towards IPV if they responded positively to at least one of the five situations, and as having a negative attitude towards IPV if they responded negatively to all five situations. Then, we aggregated values at the community level to obtain the proportion of women who support IPV. The median value was used to categorize women from the high and low proportions of communities with favorable attitudes toward IPV \| \| --- \| |
| Community media exposure | | The survey included four questions: a) the frequency of radio listening, b) the frequency of television watching, c) the frequency of reading newspapers or magazines, and d) the frequency of internet usage. Women who reported engaging in any of these activities at least once a week or more were classified as "exposed to media," while women who reported no participation were classified as "unexposed to media." At the community level, the individual scores were aggregated using the variable v001. Since the data did not follow a normal distribution, the median was used as a cutoff point. Women from communities with a proportion of media exposure equal to or above the median were classified as being from a community with high media exposure, while those from communities below the median were classified as being from a community with a low proportion of media exposure. |
| Community Education | | First, women with primary and secondary education were classified as educated, while the rest were classified as illiterate. The data was then aggregated using V001, and those with aggregated scores above the median were deemed to be from a community with a high proportion of educated individuals, while those with aggregated scores below the mean were thought to be from a community with a low proportion of educated individuals. |
| Community wealth | | Primarily, we categorize women into poor and not poor by recoding those individuals with the poorest and poorer wealth as poor and others as not poor. After that, it was aggregated, and individuals above the median were considered as from a community with a high proportion of poor individuals, and the remaining was categorized as from a community with a low proportion of poor individuals. |
